# Estimation of mosaic loss of Y chromosome cell fraction with genotyping arrays lacking coverage in the pseudoautosomal region

**DOI:** 10.1186/s12859-025-06076-6

**Published:** 2025-02-19

**Authors:** Weiyin Zhou, Wen-Yi Huang, Neal D. Freedman, Mitchell Machiela

**Affiliations:** 1 Division of Cancer Epidemiology and Genetics, National Cancer Institute, Rockville, MD USA; 2 Cancer Genomics Research Laboratory, Frederick National Laboratory for Cancer Research, Frederick, MD USA

**Keywords:** Mosaic loss of Y chromosome, Log2 R ratio, Detection, Cell fraction, Y chromosome

## Abstract

**Background:**

Mosaic loss of the Y chromosome (mLOY) in circulating leukocytes is the most frequently detected age-related chromosomal mosaic event in men. Current mLOY detection approaches use genotyping arrays and employ a phase-based approach that identifies B allele frequency (BAF) deviations in the pseudo-autosomal region (PAR) shared between the X and Y chromosome. As some widely used genotyping arrays lack sufficient probe coverage of the PAR, methods for accurately measuring mLOY utilizing the median log_2_ R ratio across the male-specific region of Y chromosome (mLRR_Y) are needed for detecting mLOY on these platforms.

**Results:**

We derived a formula from mLRR_Y to estimate the cellular fraction (CF) of cells with Y loss and validated the approach, finding high alignment with the CF estimation from female data and lab-generated qPCR data (R^2^ = 0.98). Additionally, we compared the correlation between phase-based BAF and mLRR_Y methods for CF estimation, achieving a high correlation with R^2^ > 0.80.

**Conclusion:**

Although mLRR_Y is a noisier metric for mosaic chromosomal alteration detection relative to BAF, we demonstrate mLRR_Y across non-PAR variants can accurately estimate mLOY CF, especially for high CF mLOY.

**Supplementary Information:**

The online version contains supplementary material available at 10.1186/s12859-025-06076-6.

## Background

Mosaic loss of the Y chromosome (mLOY) refers to loss of the Y chromosome in a subset of cells while the remaining cells retain a copy of the normal Y chromosome. mLOY in circulating leukocytes is the most frequently detected type of structural chromosomal mosaicism in males [1–3]. Increasing age and tobacco smoking [1, 4] are two well-established risk factors for mLOY. Varying levels of evidence have linked mLOY to a wide range of biologic and health effects [3, 5–18].

Detection of mLOY can be performed in large, existing genotyped populations by utilizing two measures of genotyping array intensity data: B allele frequency (BAF) and Log_2_ R ratio (LRR). BAF is a measure of allelic imbalance in which signal from the A allele of a variant is compared to the B allele of a variant. Deviations from homozygote or heterozygote proportions across stretches of contiguous variants are evidence of mosaic chromosomal alterations. LRR is a measure of probe signal intensity in which signals above normal are evidence for mosaic chromosomal gains and signals below normal suggest mosaic chromosomal losses. BAF signals tend to have less variability and noise relative to LRR signals.

Most current studies utilize a phase-based detection approach that examines BAF signals in the pseudo-autosomal region (PAR), focusing primarily on variants in PAR1 region, chrX:10,001- 2,781,479, GRCh38), while ignoring the much-shorter PAR2 region (chrX:56,887,903- 57,217,415, GRCh38) for detecting mLOY [19]. This phase-based BAF method relies on the assumption that BAF deviations in the PAR1 are primarily from loss of the Y chromosome, as loss of the X chromosome in male leukocytes is not observed [20]. This method leverages haplotype information to detect Y chromosome loss even in a small proportion of cells, such as less than 1%, making it the preferred method of mLOY detection when genotyping arrays have sufficient probes in the PAR1 region of the Y chromosome. The cellular fraction (CF) of cells with Y loss can be estimated using B allele frequency (BAF) deviations in the PAR1 region.

Some commonly used commercial genotyping arrays lack sufficient probe coverage in the PAR1 region, especially older array platforms like the Illumina Hap610, Hap660, and OncoArray which have fewer than 33 SNP markers (non-CNV probes) in the PAR1 region. Only a few of these SNPs are called as heterozygous loci that can be used for the phase-based BAF method. For example, using PLCO OncoArray data from the 33 SNP markers covering the PAR1 region, the minimum, median, and maximum number of heterozygous probes across 4981 male subjects was 0, 5, and 13 respectively. These numbers are insufficient for reliably calling mLOY (Supplementary Fig. 1A), necessitating alternative methods such as utilizing LRR data from the male-specific region of Y chromosome (MSY) to detect mLOY [1–3, 21]. The OncoArray has 397 probes across the MSY (Supplementary Fig. 1B), and the median LRR across these probes should produce a stable estimation for calling mLOY. As the Y chromosome in males lacks B-allele frequency (BAF) data due to its haploid state, the ability to estimate affected CF using BAF across the male-specific region of the Y chromosome is not possible. Previous studies have used qPCR data to derive regression functions for predicting the fraction of cells with Y loss [1] or have used relative terms such as decreased median LRR across the male-specific region of the Y chromosome (mLRR_Y) to indicate increased Y loss [2, 21].

In this paper, we derived a formula from measuring the mLRR_Y to estimate the proportional of cells with mLOY. We validated this formula by comparing CF estimations from female data and lab-generated qPCR data from previous study, finding high concordance. Additionally, we compared the correlation of CF estimations between phase-based BAF and mLRR_Y methods, and found a high R^2^.

## Methods

### Study population

Existing genotyping array data from the Prostate, Lung, Colorectal, and Ovarian (PLCO) screening trial were used to investigate mLOY detection and CF estimation. PLCO is a prospective cohort from a randomized multi-center trial designed to understand the effects of screening on cancer-related mortality and secondary endpoints [22].

A total of 18,756 male individuals with blood derived DNA genotyped on the Illumina Infinium Global Screening Array (GSA), 874 male individuals genotyped on the Illumina Infinium OmniExpress array (OmniEx), and 4,981 male individuals genotyped on the Illumina OncoArray were included in the mLOY analysis.

PLCO was selected for this analysis due to its availability to intramural researchers within NCI and, more importantly, its availability of genotype data from multiple array platforms. Notably, the GSA and OmniExpress arrays have sufficient variant coverage in both PAR1(N = 434 and 403 SNPs) and the male-specific region of Y chromosome (N = 1480 and 1697 SNPs), making them suitable for comparing the mLRR_Y and phase-based BAF detection methods for mLOY. In contrast, the OncoArray, while lacking adequate PAR1 variant coverage (N = 33 SNPs), included sufficient coverage of the male-specific region of the Y chromosome (N = 397 SNPs). This makes it an ideal dataset for demonstrating the importance of the mLRR_Y approach in scenarios where PAR1 coverage is limited (e.g., < 30 heterozygous variants) but coverage in male-specific region of Y chromosome is sufficient.

### Formula using mLRR_Y to estimate the proportion of cells with mLOY

For the phase-based BAF approach to detect a mosaic loss event, including loss of Y chromosome using the PAR1 region, we estimate the proportion of cells with loss using the following formula:

$${\text{Cell}}\;{\text{Fraction}}\;\left( {{\text{CF}}} \right) = {4}*{\text{Bdev}}/\left( {{1} + {2}*{\text{Bdev}}} \right)\;\left[ {{23}} \right]$$

where Bdev is the deviation from the expected BAF values of 0.5 for heterozygous loci.

For the LRR approach to estimate the abnormal cell fraction, we started with the formula:

$${\text{LRR}} = {\text{log}}_{{2}} \left( {{\text{CN}}_{{{\text{observed}}}} /{\text{CN}}_{{{\text{expected}}}} } \right).$$

For mosaic events detected in the region with an expected copy number of 2 in the normal state (e.g., autosomal or pseudo-autosomal region (PAR1)), a loss of one copy would theoretically result in an LRR of − 1. However, real data shows that LRR values are always above − 1 with a one-copy loss. Therefore, LRR needs to be rescaled to estimate the number of copies present:

$$\begin{aligned} & \left( {{\text{LRR/scale}}\;{\text{factor}}} \right) = {\text{log}}_{{2}} \left( {{\text{CN}}_{{{\text{observed}}}} {\text{/CN}}_{{{\text{expected}}}} } \right) \\ & {\text{CN}}_{{{\text{observed}} }} = {\text{CN}}_{{{\text{expected}}}} *{2}^{{({\text{LRR/scale}}\;{\text{factor}})}} . \\ \end{aligned}$$

According to Illumina’s white paper DNA Copy Number and Loss of Heterozygosity Analysis Algorithms (illumina.com), the mean LRR for a one-copy deletion (from the normal 2 copies to 1 copy) is approximately − 0.45. This value aligns with what we observe in real data. For autosomes and the PAR1 region (where CN_expected_ = 2), using 0.45 to replace the scale factor, the observed copy number is:

$${\text{CN}}_{{{\text{observed}} }} = {\text{CN}}_{{{\text{expected}}}} *{2}^{{({\text{LRR}}/{\text{scale}}\;{\text{factor}})}} = {2}*{2}^{{({\text{LRR}}/0.{45})}}$$

Using the same scale factor, the observed copy number for the Y chromosome (where CN_expected_ = 1) is:

$${\text{CN}}_{{{\text{observed}} }} = {\text{CN}}_{{{\text{expected}}}} *{2}^{{({\text{LRR}}/{\text{scale}}\;{\text{factor}})}} = {1}*{2}^{{({\text{LRR}}/0.{45})}} = {2}^{{({\text{LRR}}/0.{45})}} .$$

The final resulting model for calculating CF from mLRR_Y region is:

$${\text{CF}} = {1} - {\text{CN}} = {1} - {2}^{{({\text{mLRR}}\_{\text{Y}}/0.{45})}} .$$

**Fig. 1:**
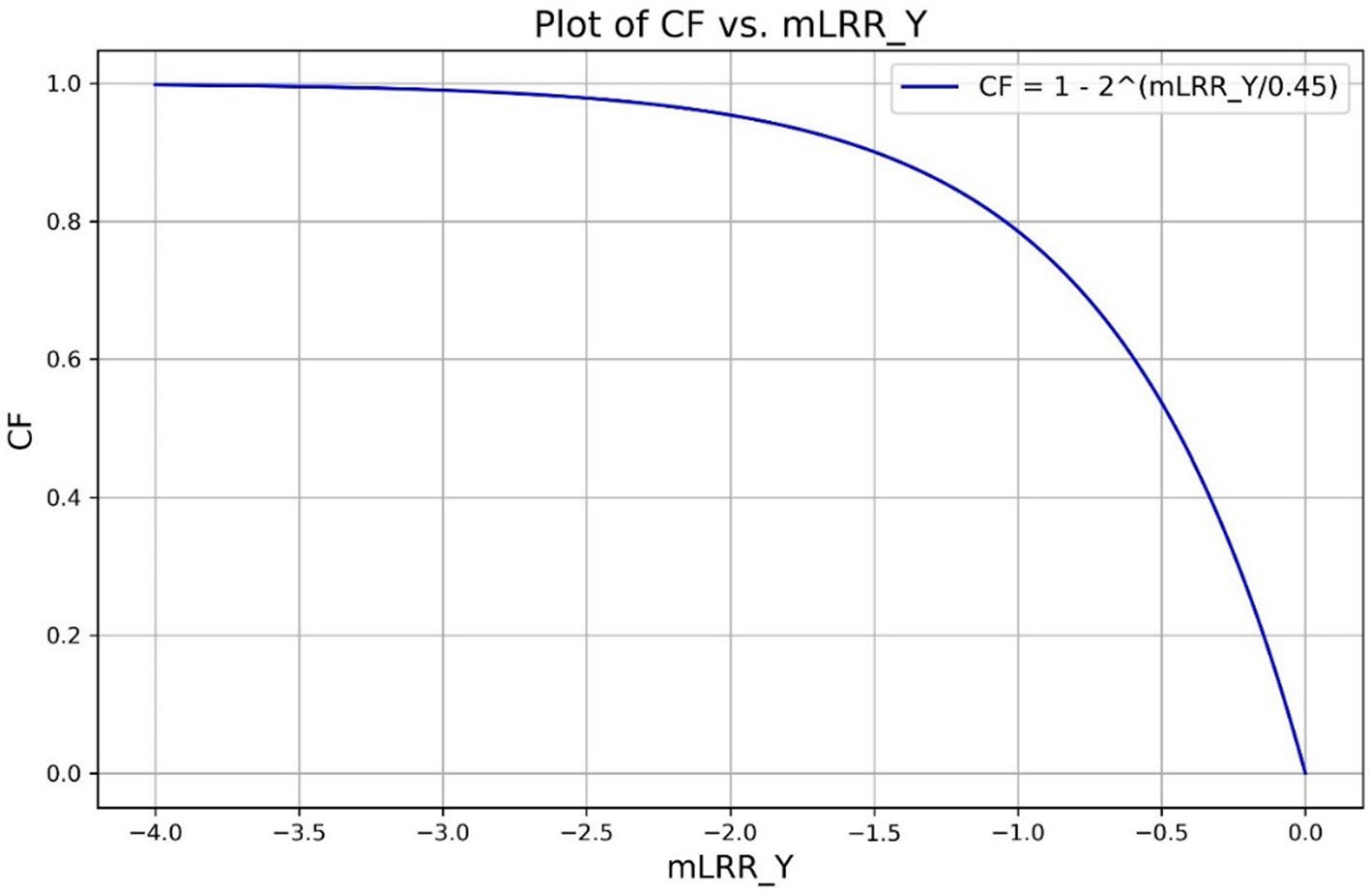
The relationship between mLRR_Y and CF using the formula CF = 1 − 2^(mLRR_Y/0.45)^

The relationship using the above formula between mLRR_Y and CF can be visualized in Fig. 1.

We also provide a reference table for quick calculation of CF from mLRR_Y in Supplementary Table 1.

### mLOY detection using the phase-based BAF approach

For mosaic loss of Y (mLOY) detection using the phase-based BAF approach, we utilized allele-specific genotyping intensities in the pseudo-autosomal region (PAR1) of the sex chromosome. This approach leverages the diploid nature of the PAR1 to detect mLOY by comparing maternal (X PAR1) and paternal (Y PAR1) allelic intensities at heterozygous sites. The proportion of cells with Y loss was estimated using BAF values in the PAR1 region.

Specifically, we employed the Mosaic Chromosomal Alterations (MoChA) WDL pipeline available at https://github.com/freeseek/mochawdl and utilized mocha.wdl v2022-05–18 (PLCO GSA) and v2022-12–21 (PLCO OmniExpress). The intensity data file (.idat) was used as the input data type, with GRCh38 as the reference genome build, and SHAPEIT4 for phasing. We identified potential mCA calls using mocha.wdl and further filtered for male samples exhibiting mLOY based on the criteria outlined at https://github.com/freeseek/mocha. These criteria included a sample call rate ≥ 0.97, a baf_auto ≤ 0.03, a minimum event size > 2MB, and a relative copy number (rel_cov) < 2.5 in the PAR1 region. Analyses were conducted on the NIH Biowulf HPC system.

## Results

### Comparisons of mLOY CF with data from female samples

**Fig. 2:**
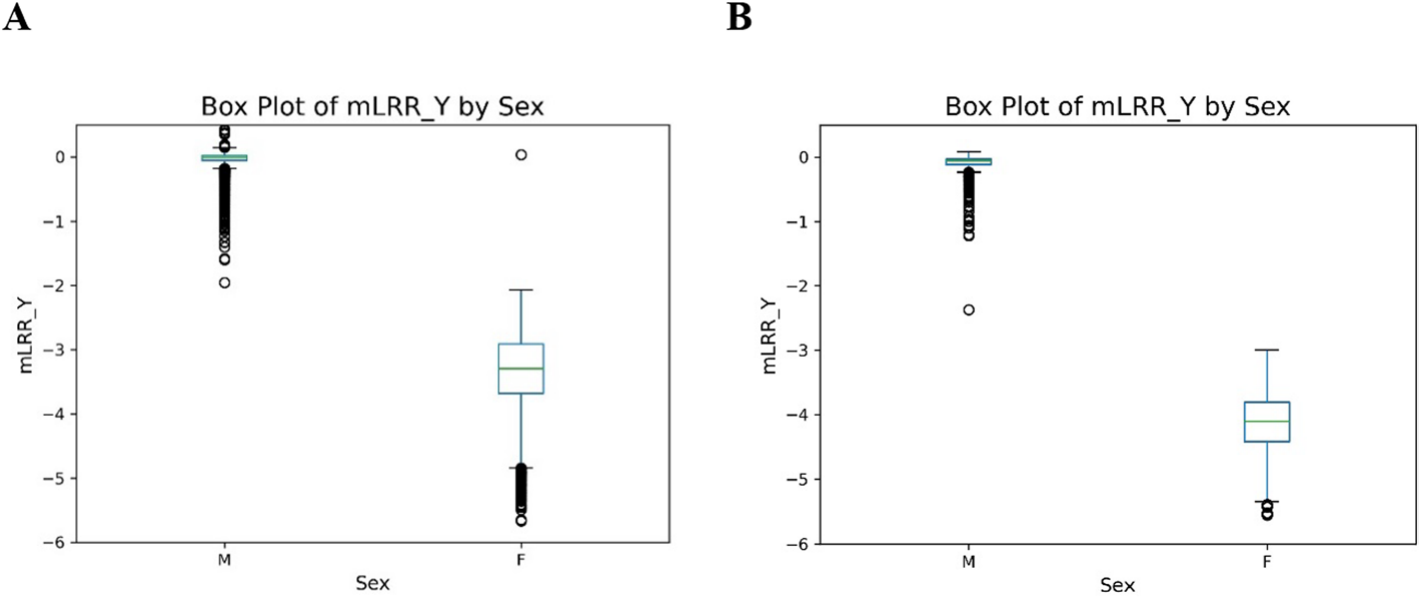
Box plots of mLRR_Y for males and females from PLCO participants genotyped on the OncoArray (**A**) and PLCO participants genotyped on the OmniExpress array (**B**)

The loss of one copy of the Y chromosome in male cells results in zero copies of the Y chromosome. Female cells also naturally have zero copies of the Y chromosome. Figure 2 shows box plots of the median mLRR_Y for male and female samples from two data sets generated from the PLCO study. In Fig. 2A, data were generated using the Illumina Infinium OncoArray chip. The median mLRR_Y for 4,981 males is − 0.007, while for 8,381 females is − 3.3. Using our formula, the mLRR_Y of − 3.3 corresponds to a copy number of 0.06, indicating a loss of 99.4% of Y cells. Since females have no Y chromosomes, this estimate is close to the 100% expectation. In Fig. 2B, data were generated using the Illumina Infinium OmniExpress chip. The median mLRR_Y for 874 males is 0.06, and for 1,113 females, it is − 4.11. Using our formula, an mLRR_Y of − 4.11 corresponds to a copy number of 0.002, indicating a loss of 99.8% of Y cells. In both instances, observed values for normal males are close to a copy number of 1 Y chromosome (0% Y loss) and observed female values are close to a copy number of 0 Y chromosomes (100% Y loss). Note that the OncoArray chip lacks probes in the PAR1 region. Using mLRR_Y approach, we are able to detect mLOY and estimate the CF using data from this array.

### Comparisons of mLOY CF with qPCR data

A prior investigation by our group [1] used an LRR threshold of − 0.15 to dichotomously call mLOY as Yes (mean LRR_Y ≤ − 0.15) and No (mean LRR_Y > − 0.15). We developed a model to predict the mLOY cell fraction using a quadratic regression model to fit the average qPCR ratio and mean LRR data pairs, with mean LRR as the predictor variable and average qPCR ratio as the response variable. Only the data points from subjects having consensus event calls between qPCR and chip data for Y chromosome loss and normal, with coefficient of variation (CV) ≤ 10% from qPCR data, were used to generate the prediction model (*n* = 98). For each mean LRR, the corresponding copy number can be predicted by inserting the mean LRR into the quadratic equation. The CF for mLOY equaled 1 minus the average Y chromosome signal ratio. For example, a mean LRR of − 0.15 corresponded to a frequency of Y chromosome loss of 22.7%. Using the current formula (CN = 2^(−0.15/0.45)^ = 0.794; CF = 1 − 0.794 = 0.204) provides a mLOY CF of 0.204 which is highly similar to that of the CF derived from the qPCR model (0.227). Likewise a mean LRR of − 0.5 corresponded to a qPCR estimated frequency of Y chromosome loss of 52.5%. Using the current formula (CN = 2^(−0.5/0.45)^ = 0.463; CF = 1 − 0.463 = 0.537) provides a mLOY CF of 0.537, which again is highly similar to that of the CF derived from the qPCR model (0.525). For the data set used in the prior investigation of mLOY [1], the mean_LRR values for mLOY ranged from − 0.15 to − 1.384. Using points in this range, the linear correlation for the CF derived from both methods is highly correlated with R^2^ = 0.98 (Supplementary Table S2, Supplementary Fig. 2).

### Correlation of mLOY CFs between phase-based BAF and mLRR_Y approaches

We next tested the correlation between CF values estimated from the phase-based BAF approach (CF_BAF_) utilizing PAR1 variants with CF values from the mLRR_Y approach (CF_mLRR_Y_) from male subjects genotyped on Illumina arrays with probes spanning both the PAR1 and the male-specific Y regions. mLOY was detected using the phase-based BAF approach and mLRR_Y was calculated. Phase-based BAF mLOY calls with mLRR_Y greater than 0 were removed as CF_mLRR_Y_ would be positive for these men. Likewise, men with mLRR_Y less than 0, but not called by the phase-based BAF approach were not evaluated as no CF_BAF_ were available.

**Fig. 3:**
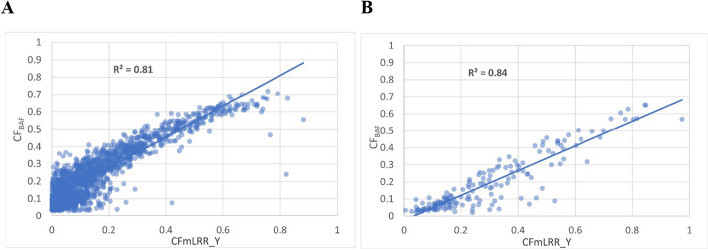
Correlation between mLOY CF estimates using the phased PAR1 (CF_BAF_) and calculated mLRR_Y (CF_m__LRR_Y_). The CF_m__LRR_Y_ on the X axis represent the CF estimations from mLRR_Y. The CF_BAF_ on the Y axis represents the CF estimates for the phase-based BAF method using PAR1 variants. **A** Represents data from the PLCO GSA chip, while **B** represents data PLCO OmniExpress chip

For the first test set, we utilized data from 18,756 existing blood-derived DNA male samples from the Prostate, Lung, Colorectal and Ovarian Cancer Screening Trial (PLCO) that were genotyped on the Illumina Global Screening Array (GSA) [22]. We identified 1,670 men with mLOY using the phase-based BAF approach and with mLRR_Y values less than 0. The CF from both approaches was calculated. The correlation between CF_BAF_ and CF_mLRR_Y_ yielded an R^2^ of 0.81 indicating high correlation (Fig. 3A).

**Fig. 4:**
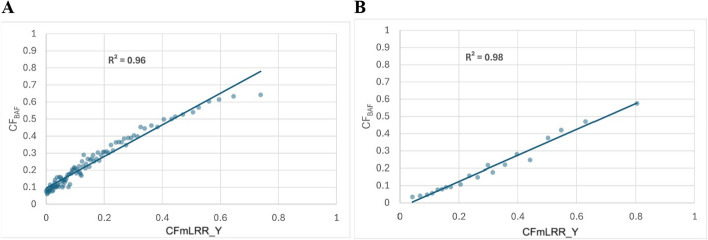
Correlation of abnormal cell fraction between mLOY calls using the phase-based BAF PAR1 (CF_BAF_) and calculated mLRR_Y (CF_mLRR_Y_) from sorted mLRR_Y data split into equal bins. The CF_mLRR_Y_ on the X axis represent the median of CF estimations from mLRR_Y in each bin. The CF_BAF_ on the Y axis represents the median of CF estimates for the phase-based BAF method using PAR1 variants in each bin. **A** Represents data from 100 bins of the PLCO GSA data, while **B** represents data from 20 bins of the PLCO OmniExpress data

We further divided mLRR_Y values from the above 1,670 men with mLOY into 100 bins. For each bin, the minimum, maximum, median, and mean of CF calculated from mLRR_Y values (CF_mLRR_Y_) were determined. Additionally, the same set of values of CF calculated from the phase-based BAF approach (CF_BAF_) were determined for the same bins. The correlation between median CF_BAF_ and median CF_mLRR_Y_ yielded an R^2^ of 0.96 (Fig. 4A and Supplementary Table 3A).

In the second test set, we utilized existing data from 874 PLCO blood-derived DNA male samples genotyped on the Illumina OmniExpress array. We identified 164 men with mLOY using the phase-based BAF method and with mLRR_Y less than 0. The abnormal cell fraction from both approaches was calculated. The R^2^ of estimations from CF_BAF_ and CF_mLRR_Y_ was 0.84 (Fig. 3B). We also divided the 164 men with mLOY into 20 mLRR_Y bins. For each bin, the minimum, maximum, median, and mean of CF calculated from mLRR_Y values (CF_mLRR_Y_) were calculated. The same set of values of CF calculated from BAF approach (CF_BAF_) were calculated for the same bins. The correlation between median CF_BAF_ and median CF_mLRR_Y_ yielded an R^2^ of 0.98 (Fig. 4B and Supplementary Table 3B).

### Instances where mLRR_Y mLOY calling outperforms the phase-based BAF approach

**Fig. 5:**
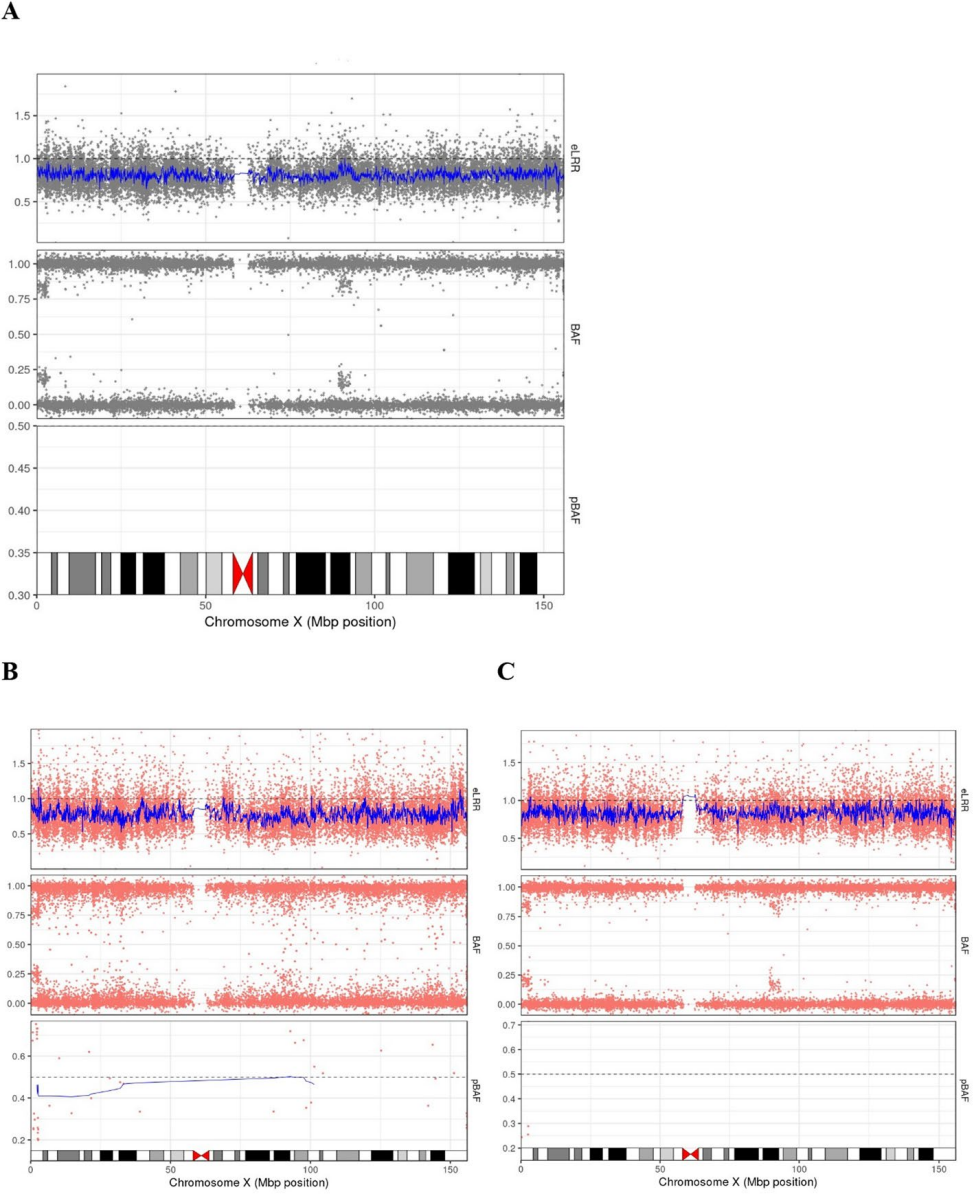
Example mLOY plots using the phase-based BAF approach, which utilizes variants in the PAR1 region (chrX:10,001–2,781,479, GRCh38). The plots illustrate instances of failed detection or underestimated cell fractions (CF) of mLOY. Grey regions represent areas without detected events, while orange regions indicate detected events. **A** An example where mLRR_Y identified a mLOY event that the phase-based BAF approach failed to detect. **B** An instance of high CF mLOY with an underestimated abnormal cell fraction. **C** Another example of high CF mLOY with an underestimated abnormal cell fraction

Reduced sensitivity and underestimated abnormal cell fraction are the issues for men with high CF mLOY using the phase-based BAF detection approach. The phase-based BAF approach can fail to detect some men with high CF mLOY due to a limited number of available heterozygous calls (Fig. 5A). As only a small proportion of true heterozygous loci are correctly called as AB genotype, this can also lead to an underestimation of the abnormal cell fraction. In Fig. 5B, the Bdev is 0.234, corresponding to a CF of 0.638. Using mLRR_Y, the CF estimate is higher at 0.652, corresponding to a Bdev of 0.242. In Fig. 5C, the Bdev is 0.231, corresponding to a CF of 0.633. Using mLRR_Y, the CF is again higher at 0.719, corresponding to a Bdev of 0.281. From the BAF plot in the middle BAF panel of both Fig. 5B, C, the values 0.242 and 0.281 provide a better estimation of Bdev from the true data. Additionally, although the two BAF bands in the middle panel of Fig. 5C shows a larger split than in Fig. 5B, its Bdev estimation from the phase-based BAF method is smaller. This is because almost all heterozygous loci in Fig. 5C are called as homozygous and excluded from phasing, mCA detection, and CF estimation, as shown by the very few loci in the phased BAF (pBAF) of the bottom panel. Only those with lower BAF values in the region are correctly called AB, leading to their inclusion in phasing, mCA detection, and CF estimation. These lower BAF values cause an underestimation of the mLOY CF.

## Discussion

Our presented mLRR_Y approach utilizes the intensity of genotyped probes in the male-specific non-PAR region of the Y chromosome to identify mLOY and estimate abnormal cell fraction (CF). This approach accurately estimates CF and does not rely on the BAF deviation of heterozygous probes in the PAR1 region, providing an alternative approach to estimate the percentage of cells with Y loss in genotyping arrays lacking sufficient coverage in the PAR1 region.

We tested the correlation between CF estimations obtained using the phase-based BAF and mLRR_Y approaches in male mLOY participants genotyped on Illumina chips and observed a high correlation (R^2^ > 0.8). The high correlation was validated using the mLRR_Y CF estimation calculated using genotyped probes in the male-specific region of Y chromosome. This observation also provides evidence that mLOY detected from BAF in the PAR1 region reflects mLOY and is not an artifact related to the X chromosome loss in males. Loss of the X chromosome is not observed in males [20].

There were instances where the two mLOY detection methods differed. We noted cases where the phase-based BAF detection approach detected mLOY, but the mLRR_Y was greater than zero, indicating no mosaic loss of Y in the LRR signal. This discrepancy is generally due to lower cell fraction mLOY events that are detectable using phase-based BAF, but for which the noisier LRR signal is unable to identify. For this reason, the phase-based BAF approach is more sensitive for calling mLOY with lower cell fractions. We also noted instances where mLRR_Y detected a mLOY event but for which the phase-based BAF approach did not detect an event. The phase-based BAF approach is susceptible to missing high cell fraction mLOY as the heterozygous loci (AB genotypes) needed as input for the phase-based BAF approach can be misclassified as homozygous (AA/BB genotypes). This can lead to missed detection of mLOY and underestimated abnormal cell fractions.

There are notable limitations of the mLRR_Y calling approach. First, mLRR_Y tends to be noisier than BAF, this potentially results in a less accurate mLOY CF estimation for low to medium levels of mLOY compared to the phase-based BAF approach, although our investigation shows high concordance of CF in ranked mLRR_Y bins with phase-based BAF CF estimates. Second, as the LRR signals contain more noise, the mLRR_Y calling approach is less sensitive for detecting mLOY in low cell fractions. The lowest CF detectable using the mLRR_Y approach is 20.6%, corresponding to an mLRR_Y cutoff of − 0.15 for mLOY, whereas the phase-based BAF method can detect cell fractions as low as 1.47% in the PLCO GSA dataset and 2.1% in the PLCO OmniEx dataset. Conversely, the mLRR_Y approach can identify mLOY in higher CF samples, detecting fractions as high as 95.1% in the PLCO OncoArray dataset, and 88% and 97.4% in the PLCO GSA and PLCO OmniEx datasets, respectively. In contrast, the highest CFs detected using the phase-based BAF method are 72% and 65% in the PLCO GSA and PLCO OmniEx datasets. This is a feature of the mLRR_Y calling approach that should be noted in investigations that implement this method as higher CF mLOY can have different associations with disease outcomes than lower CF mLOY [6].

Given these differences, the two methods complement each other effectively: mLRR_Y provides more accurate CF estimations for high-level mLOY, while the phase-based BAF method demonstrates superior sensitivity for detecting very low-level mLOY. When there are enough heterozygous variants in both PAR1 and male-specific region of Y chromosome, employing both methods together offers a robust and comprehensive approach to mLOY detection, maximizing accuracy across the entire spectrum of cell fractions.

Importantly, the mLRR_Y method serves as a valuable alternative when PAR1 coverage is insufficient (e.g., fewer than 30 heterozygous variants) but MSY coverage is adequate (e.g., ≥ 30 probes). Unlike the phase-based approach, mLRR_Y leverages all probes, not just heterozygous variants, as it does not depend on phased data. This reduces coverage requirements, making mLRR_Y a practical and effective option for mLOY detection in datasets with limited PAR1 coverage.

## Conclusions

For arrays lacking sufficient variants in the PAR1 region, we derived a formula from mLRR_Y to estimate the proportion of cells with Y loss (CF_mLRR_Y_ = 1 − 2^(mLRR_Y/0.45)^). We validated this formula and found the CF estimates align with those from female data and lab-generated qPCR data. Additionally, we compared the correlation of CF estimates between phase-based BAF and mLRR_Y methods, achieving a high correlation. While mLRR_Y tends to be noisier signal than BAF, we find that mLRR_Y can be used to estimate mLOY cell fractions with high accuracy.

## Supplementary Information

Supplementary Material 1.

## Data Availability

Genotyping array data supporting the findings of this study are available through the Prostate, Lung, Colorectal, and Ovarian (PLCO) Screening Trial and can be accessed via the dbGaP repository with accession number phs001286.v3.p2. Additional data is provided within the manuscript and supplementary information files.

## Abbreviations

BAF: B allele frequency
Bdev: Deviation from the expected BAF values of 0.5 for heterozygous loci
CF: Cellular fraction
CF_BAF_: CF calculated from BAF approach
CF_mLRR_Y_: CF calculated from mLRR_Y values
CN: Copy number
GSA: Global Screening Array
LRR: Log_2_ R ratio
MSY: Male-specific region of Y chromosome
mLOY: Mosaic loss of Y chromosome
mLRR_Y: Median log_2_ R ratio across the male-specific region of Y chromosome
MoChA: Mosaic Chromosomal Alterations
pBAF: Phased B allele frequency
PAR: Pseudo-autosomal region
PAR1: Pseudo-autosomal region 1 (chrX: 10,001- 2,781,479, GRCh38)
PLCO: The Prostate, Lung, Colorectal and Ovarian Cancer Screening Trial
